# The correlation between kidney volume and measured glomerular filtration rate in an Asian ADPKD population: a prospective cohort study

**DOI:** 10.1186/s12882-021-02392-0

**Published:** 2021-05-15

**Authors:** Bunyong Phakdeekitcharoen, Watcharapong Treesinchai, Pornphan Wibulpolprasert, Sarinya Boongird, Pinkael Klytrayong

**Affiliations:** 1 Division of Nephrology, Department of Medicine, Faculty of Medicine, Ramathibodi Hospital, Mahidol University, 10400 Bangkok, Thailand; 2 Department of Diagnostic and Therapeutic Radiology, Faculty of Medicine, Ramathibodi Hospital, Mahidol University, Bangkok, Thailand

**Keywords:** Autosomal dominant polycystic kidney disease (ADPKD), Measured GFR (mGFR), Iohexol, Total kidney volume (TKV), Ultra performance liquid chromatography (UPLC)

## Abstract

**Background:**

Autosomal dominant polycystic kidney disease (ADPKD) is the most common hereditary kidney disorder that leads to end stage renal disease (ESRD). Cyst expansion in ADPKD is strongly associated with the decline in renal function. However, the correlation between total kidney volume (TKV) and glomerular filtration rate (GFR) at an early stage has not been well demonstrated. There is growing evidence that utilization of estimated GFR (eGFR) may induce misleading information in a population with near normal renal function. Therefore, a more accurate method is essential.

**Methods:**

A prospective cohort of ADPKD patients was conducted with clinical data and laboratory collection. Measured GFR (mGFR) was assessed by iohexol plasma clearance method using ultra performance liquid chromatography. eGFR was calculated using the CKD-EPI equation. Kidney volumes were evaluated using MRI imaging protocol.

**Results:**

Thirty two patients completed the study. The mean age was 56 years old. The mean initial mGFR was 83.8 mL/min/1.73m^2^. The mean change in mGFR per year was –2.99 mL/min/1.73m^2^/year. The mean initial height-adjusted TKV (htTKV) was 681.0 mL/m. The mean percentage change in htTKV per year (%ΔhtTKV/y) was 4.77 %/year. mGFR had a better association with clinical parameters than eGFR. Initial mGFR was significantly and inversely correlated with initial htTKV and age. The percentage change in mGFR per year was significantly and inversely correlated with the %ΔhtTKV/y and 24-hr urine albumin. The %ΔhtTKV/y was significantly correlated with initial htTKV.

**Conclusions:**

Our studies demonstrated that mGFR using iohexol is a more reliable and accurate method than eGFR for evaluating GFR changes in the early stages of ADPKD patients. There is a strong inverse correlation between kidney volume and mGFR in an Asian ADPKD population. The initial htTKV is a good predictor of kidney volume progression. The %ΔhtTKV/y is a good early surrogate marker for the decline in renal function. 24-hr urine albumin is also a good indicator for renal progression.

## Background

Autosomal dominant polycystic kidney disease (ADPKD) is the most common hereditary kidney disorder that leads to end stage renal disease (ESRD) [1, 2]. Clinical presentation varies widely, including hypertension, hematuria, proteinuria and renal insufficiency [3]. It is characterized by gradual renal enlargement and cyst growth prior to loss of renal function [4]. The average age of entry into ESRD is 57 years [5]. The kidney function remains stable in ADPKD patients for years, followed by a sharp decline in glomerular filtration rate (GFR) once a critical renal size is reached [6, 7]. Clinical parameters associated with decline in kidney function in ADPKD patients have been gradually reported [8–10]. However, the lack of a sensitive measure of disease progression in the early stages of ADPKD has caused problems in the development of prevention and of therapeutic agents [11–13].

Imaging methods that reliably and accurately measure total kidney volume (TKV) in ADPKD patients have been developed utilizing magnetic resonance imaging (MRI) and computerized tomography [14–16]. The Consortium of Radiologic Imaging Studies of Polycystic Kidney Disease (CRISP) study has demonstrated that the expansion of cysts in patients with ADPKD is strongly associated with the decline in renal function [14]. Higher rates of kidney enlargement are associated with a more rapid decline in renal function. However, the correlation between TKV and the accuracy of GFR changes at an early stage has not been well demonstrated. The results of clinical trials have shown no beneficial effects of sirolimus or everolimus therapy against progressive GFR decline in two large cohorts of ADPKD patients using estimated GFR by calculation, namely the Chronic Kidney Disease Epidemiology Collaboration (CKD-EPI) and the Modification of Diet in Renal Disease (MDRD) equations, that is based on serum creatinine levels represented as a marker of glomerular filtration [12, 13]. These formulas have been constantly evaluated and there is growing evidence that their utilization may induce misleading information, especially in population with normal or near normal renal function [17–21]. The authors in previous studies have suggested that in early stages of ADPKD, the variability in serum creatinine levels may be explained by creatinine production related to muscle mass or protein intake rather than glomerular filtration [22]. Therefore, direct measurements of GFR by gold-standard techniques depended on the use of exogenous markers such as inulin, iohexol or radio-labeled tracers, which would be essential in order to assess the accuracy of GFR decline in the studied population [23–25].

In this study, we compared the efficacy between eGFR using CKD-EPI and mGFR using iohexol plasma clearance for evaluating GFR changes in the early stages of patients with ADPKD and investigated the relationship between clinical parameters, total kidney volume and renal function and identified the risk factors associated with the progression in kidney structure and function decline in the Thai ADPKD population.

## Methods

### Study population

A prospective cohort study was conducted, with test subjects being patients under the care of the nephrology division, Department of Medicine, Ramathibodi Hospital, during 2015–2019. The inclusion criteria are as follows: diagnosis of ADPKD (by family history, the Ravine’s criteria [26], and genetic analysis [27]), age more than 18 years, clinically stable, eGFR ≥ 30 mL/min/1.73m^2^ by CKD-EPI and willing to join the study. The exclusion criteria included undergoing transplantation, contraindication for MRI, pregnancy, and a high mortality disease such as cancers. At each visit, the medical history, adverse events, medical changes, and hospitalizations were recorded. Other clinical data were prospectively collected including: age of enrollment, weigh, height, body mass index (BMI), mean systolic blood pressure (SBP), mean diastolic blood pressure (DBP), serum creatinine (sCr), serum uric, fasting blood sugar, and LDL-cholesterol. 24-h urine protein and 24-h urine albumin were collected after being enrolled in the study for 4 weeks. Kidney volume and GFR were measured annually. All methods were performed in accordance with the relevant guidelines and regulations of the institute. Written informed consent was obtained from all patients. The study was approved by the Institutional Ethics Committee on Human Rights Related to Research Involving Human Subjects of the Ramathibodi Hospital, Mahidol University.

### GFR measurements

mGFR was determined by the plasma clearance of iohexol. Briefly, iohexol was administered as Omnipaque® 300 mg I/mL (Amersham Health, South Plainfield, NJ, USA). Venous blood samples were collected at four time points (120, 180, 240, and 300 min) after a single 5-ml intravenous injection of iohexol. Plasma concentration of iohexol was determined by ultra performance liquid chromatography (UPLC). The UPLC analytical system was performed with Agilent technologies (Agilent Technologies, Wilmington, DE, USA). The system was connected to the autosampler UV detector. The manager software was ChemStation. The intra-assay and inter-assay variant of UPLC were 0.94 and 2.44 %, respectively. A curve was obtained expressing iohexol elimination. We calculated measured GFR by using the one-compartment model, often referring as the slope-intercept technique. The one-compartment clearance, *Cl*_*1*_, is the ratio between the injected amount of iohexol dose and the area under the slow-compartment (final monoexponential part) of plasma concentration curve. Since the *Cl*_*1*_ did not include the whole area of plasma concentration curve, the mathematical correction for the first fast exponential curve is needed. Herein, we used the correction equation proposed by Brochner-Mortensen (BM) to calculate the measured GFR [28]. GFR from BM correction (mL/min) = (0.990778× *Cl*_*1*_) − (0.001218× *Cl*_*1*_^*2*^). All mGFR results were adjusted for body surface area.

The morning of each iohexol clearance study, serum creatinine concentration was measured with enzymatic method using an automatic device (Architec c16000, Abbott core laboratory, Illinois, USA). eGFR was calculated using the CKD-EPI equation [29].

### MRI imaging protocol

All renal MRI imaging were performed on a 3.0-T MRI scanner (Ingenia MR system; Philips, Best, Netherland). The MRI protocol included sshT2W, IDEAL, non-contrast enhanced T1W, and dynamic contrast enhanced MRI sequences. Following this acquisition of pre-gadolinium images, 0.1 mmol/kg gadobutrol (Gadovist; 1.0 mmol/mL) was injected at 1mL/s. The post-gadolinium dynamic imaging was acquired in three initial phase (25, 40, and 60 s after contrast administration), and then after a delay of 3, 5, and 10 min, with a bolus tracking technique and fat saturation. Kidney volumes were measured by manually tracing the kidney contours using volume analysis solfware implemented on an Advantage Windows Workstation (4.4, GE Healthcare, Buc, France) as previously prescribed in the study protocol [30]. The sshT2W images were used by the experience radiologists for measuring kidney volumes since this non gadolinium MRI sequence provided reliable kidney volume measurement [30].

### Data and Statistical Analysis

The study was designed as a prospective cohort study. Statistics were analyzed with SPSS V 18.0. Numerical data were shown with mean ± SD or median (range). Pearson’s correlation coefficients were used to examine the association among eGFR or mGFR, the percentage change in eGFR or mGFR per year (%ΔeGFR/y or %ΔmGFR/y), the percentage change in height-adjusted total kidney volume per year (%ΔhtTKV/y), and selected clinical variables. Univariate and multivariate regression analyses were used to determine independent predictors of baseline eGFR or mGFR, %ΔeGFR/y or %ΔmGFR/y, and %ΔhtTKV/y. Two-tail *p* values of < 0.05 were considered to indicate a statistically significant difference.

## Results

### Patients’ characteristics

Initially, there were 41 ADPKD patients included in the study. One patient was excluded from the study due to nephrotic syndrome and eight were dropped off due to incomplete follow up. Thirty two (25 female and 7 male) patients completed the study. The mean duration of follow-up time was 1.79 ± 0.58 years. Table 1 shows the baseline characteristics and contemporaneous measurements of the patients. There is no statistical difference in clinical parameters between complete and incomplete study group. In the complete study group, the mean age was 56.3 ± 12.7 years old. The mean baseline systolic and diastolic blood pressure was 130.6 ± 15.5 and 79.2 ± 8.7 mmHg, respectively. Out of 32 patients, 24 (75 %) had hypertension and 2 (6.3 %) developed hypertension before the age of 35 years old. All hypertensive patients were advised to take angiotensin receptor blockers (ARBs) or angiotensin converting enzyme inhibitors (ACE inhibitors) unless they had an allergy or intolerance to them. Of the 24 hypertensive patients, 19 (59.4 %) took ARBs or ACE inhibitors and 12 (37 %) had calcium channel blockers. Four (12.5 %) patients had a history of gross hematuria and 7 (21.9 %) experienced a condition of infected cysts. No patient had undergone cyst aspiration or sclerotherapy for large kidney cysts during the study period.

**Table 1:** Baseline characteristics and contemporaneous measurements of the patients in this study

	Complete study (*N* = 32)	Incomplete study (*N* = 8)
Age (years)	56.3 ± 12.7	55.3 ± 11.8
Female (N, %)	25 (78.1 %)	5 (62.5 %)
BMI (Kg/m^2^)	23.7 ± 3.2	23.8 ± 2.9
BMI > 25 (N, %)	10 (31 %)	3 (37.5 %)
SBP (mmHg)	130.6 ± 15.5	130.9 ± 15.6
DBP (mmHg)	79.2 ± 8.7	79.5 ± 8.8
History of smoking (N, %)	5 (15.6 %)	1 (12.5 %)
Hypertension (N, %)	24 (75 %)	6 (75 %)
Hypertension before 35 year old	2 (6.3 %)	1 (12.5 %)
Hypertension after 35 year old	22 (68.8 %)	5 (62.5 %)
Antihypertensive medication (N, %)		
ARB or ACE inhibitors	19 (59.4 %)	5 (62.5 %)
Calcium channel blockers	12 (37.5 %)	3 (37.5 %)
History of gross hematuria from ruptured cysts (N, %)	4 (12.5 %)	1 (12.5 %)
History of infected cysts (N, %)	7 (21.9 %)	2 (25 %)
Fasting blood sugar (mg/dL)	96.6 ± 9.0	95.4 ± 8.6
LDL cholesterol (mg/dL)	109.2 ± 28.1	110.3 ± 29.8
Serum uric acid (mg/dL)	6.01 ± 1.26	6.11 ± 1.29
Serum albumin (g/L)	37.7 ± 6.9	37.6 ± 5.1
Serum creatinine (mg/dL)	0.94 ± 0.39	0.96 ± 0.36
eGFR: CKD-EPI (mL/min/1.73 m^2^)	80.3 ± 24.9	79.8 ± 24.8
mGFR (mL/min/1.73 m^2^)	83.8 ± 26.1	80.7 ± 24.0 (*N* = 2)
htTKV (mL/m)	681.0 ± 476.3	670.9 ± 452.7 (*N* = 3)
Mayo imaging classification^a^		
1 A (N, %)	8 (25 %)	1 (33.3 %)
1B (N, %)	11 (34.4 %)	1 (33.3 %)
1 C (N, %)	9 (28.1 %)	1 (33.3 %)
1D (N, %)	4 (12.5 %)	0
1E (N, %)	0	0
24-hr urine protein (mg/day/1.73 m^2^)	205.5 ± 174.0	194.5 ± 177.5 (*N* = 2)
24-hr urine albumin (mg/day/1.73 m^2^)	79.0 ± 120.2	77.1 ± 85.2 (*N* = 2)

Data presented as mean ± SD unless otherwise indicated

*BMI* body mass index; *SBP* systolic blood pressure; *DBP* diastolic blood pressure; *ARB* angiotensin receptor blocker; *ACE inhibitor* angiotensin converting enzyme inhibitor; *LDL cholesterol* low density lipoprotein cholesterol, *eGFR* estimated glomerular filtration rate; *CKD-EPI* Chronic Kidney Disease Epidemiology Collaboration; *mGFR* measured glomerular filtration rate; *htTKV* height-adjusted total kidney volume

^a^Reference [31]

**Fig. 1:**
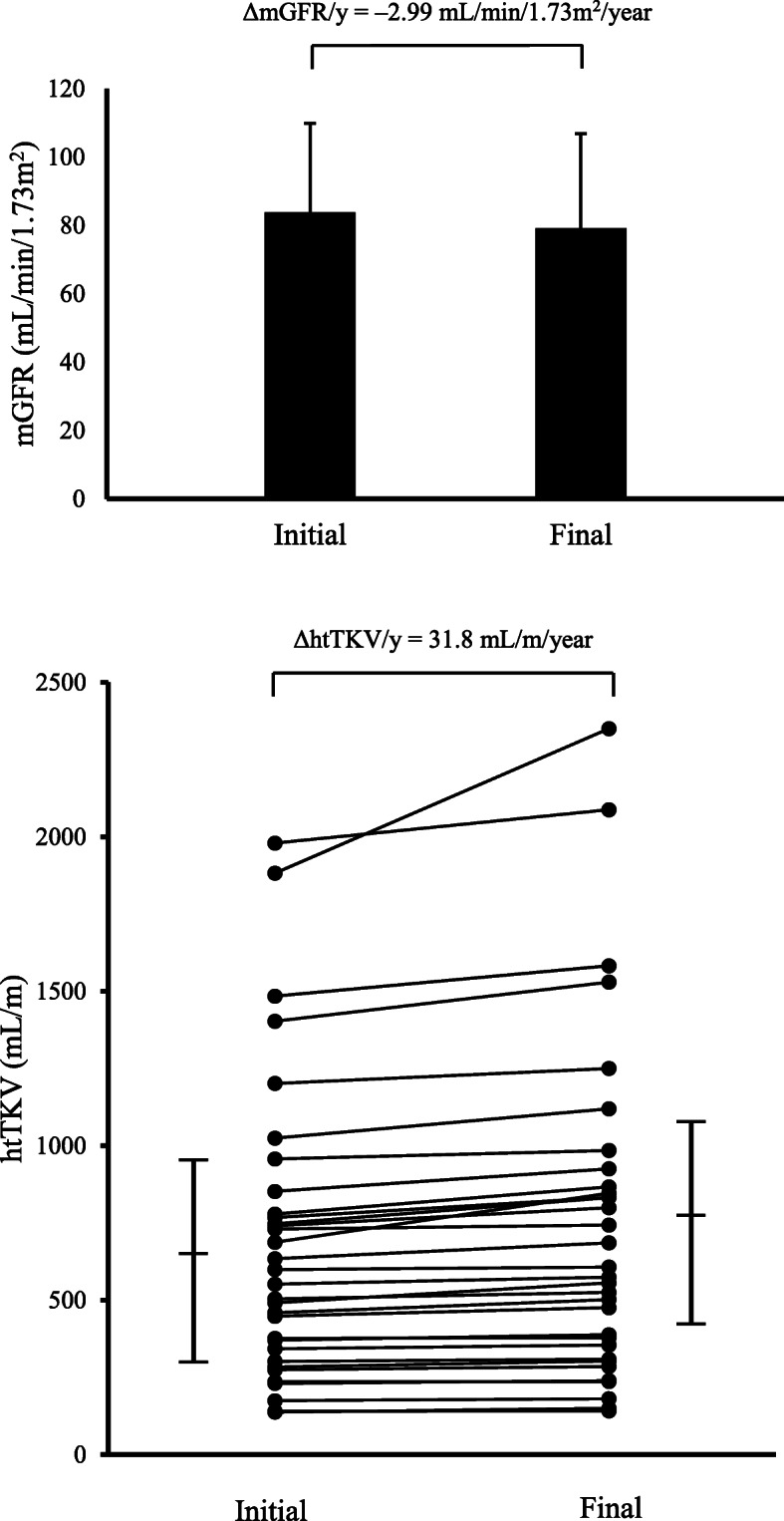
**a** The changes in mGFR and **b** htTKV in 32 ADPKD patients over the study period. mGFR; measured glomerular filtration rate, ΔmGFR/y; the mean change in mGFR per year; htTKV; height-adjusted total kidney volume; ΔhtTKV/y, the mean change in htTKV per year

The mean (± SD) baseline serum creatinine was 0.94 ± 0.39 mg/dL. The mean initial eGFR by CKD-EPI was 80.3 ± 24.9 mL/min/1.73m^2^.The mean initial mGFR was 83.8 ± 26.1 mL/min/1.73m^2^. The mean change in eGFR and mGFR per year (ΔeGFR/y & ΔmGFR/y) was –2.91 ± 2.75 and –2.99 ± 2.86 mL/min/1.73m^2^/year, respectively. The mean percentage change in eGFR and mGFR per year (%ΔeGFR/y & %ΔmGFR/y) was –4.14 ± 4.87 and –4.21 ± 4.45 %/year, respectively (Fig. 1). The mean initial TKV was 1083.4 ± 738.7 mL and the mean initial htTKV was 681.0 ± 476.3 mL/m. The mean change in htTKV per year (ΔhtTKV/y) was 38.1 ± 61.9 mL/m/year and the mean percentage change in htTKV per year (%ΔhtTKV/y) was 4.77 ± 3.90 %/year (Fig. 1). According to the Mayo clinic ADPKD classification scheme [31], patients were divided into 5 groups by htTKV and age (1A to 1E, low risk to high risk); most patients in this study were identified as group 1A, 1B and 1C (Table 1). All of the patients remained in the same class over the duration of the study. The mean 24-hr urine protein and 24-hr urine albumin were 205.5 ± 174.0 and 76.2 ± 111.2 mg/day/1.73 m^2^, respectively.

### Comparison between eGFR and mGFR for clinical correlation

**Fig. 2:**
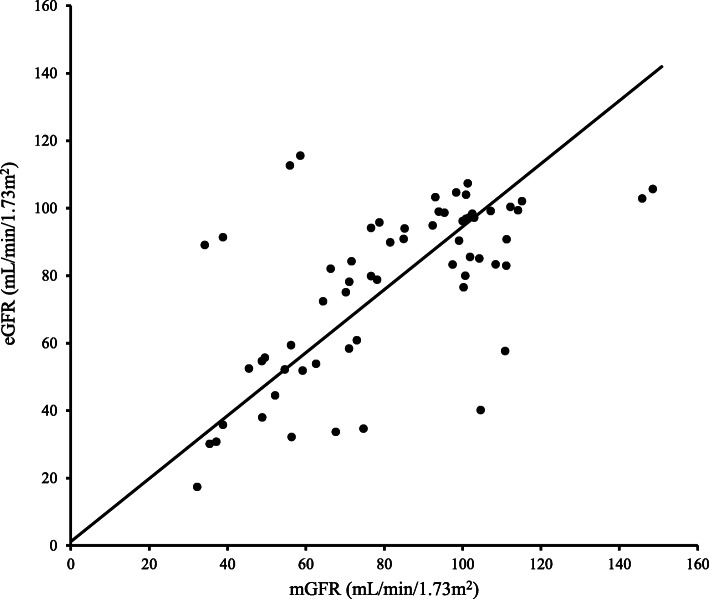
The relationship between eGFR by CKD-EPI and mGFR by iohexol plasma clearance in 32 ADPKD patients over the study period using all data points of each patient. eGFR is significantly correlated with mGFR (*y* = 0.933*x* + 0.11, *r* = 0.718, *p* < 0.001). mGFR; measured glomerular filtration rate, eGFR; estimated glomerular filtration rate, CKD-EPI; chronic kidney disease epidemiology collaboration

Renal function evaluated by eGFR had a strong association with mGFR (*r* = 0.718, *p* < 0.001). The equation was as follows: *y* = 0.933*x* + 0.11 (Fig. 2). However, when compared the clinical correlation between eGFR and mGFR, we found that mGFR had a better association with clinical parameters. Table 2 demonstrated that initial mGFR had a better association with clinical parameters compared to initial eGFR. Initial mGFR was significantly associated with 4 clinical parameters, including age, initial htTKV, 24-hr urine protein and 24-hr urine albumin, while initial eGRF was significantly associated with only 2 clinical parameters (age and initial htTKV). Moreover, in these two significant clinical parameters, initial mGFR had a better association compared to initial eGFR (*p* age: 0.015 vs. 0.041, *p* initial htTKV: <0.001 vs. 0.003). Table 3 also confirmed the finding that mGFR had a better association with clinical parameters compared to eGFR. Table 3 demonstrated that the percentage change in measured GFR per year (%ΔmGFR/y) had a better association with clinical parameters compared to the percentage change in estimated GFR per year (%ΔeGFR/y). %ΔmGFR/y was significantly associated with 4 clinical parameters, including initial mGFR, 24-hr urine protein, 24-hr urine albumin and percentage change in height-adjusted total kidney volume per year (%ΔhtTKV/y), while %ΔeGFR/y was significantly associated with only one clinical parameter (initial eGFR). Furthermore, in this significant clinical parameter, %ΔmGFR/y had a better association compared to %ΔeGFR/y (*p* initial mGFR vs. *p* initial eGFR: 0.006 vs. 0.030).

**Table 2:** The association of initial eGFR and initial mGFR on different clinical parameters

Clinical parameters (*N* = 32)	initial eGFR			initial mGFR					
	**Univariate analysis**			**Univariate analysis**			**Multivariate analysis**		
	*r*	95 % CI	*p*	*r*	95 % CI	*p*	*r*	95 % CI	*p*
Age (year)	–0.364	**–**1.390 to **–**0.032	0.041*	**–**0.427	–1.568 to –0.184	0.015*	**–**0.346	**–**1.279 to **–**0.142	0.016*
BMI (Kg/m^2^)	–0.164	–4.198 to 1.609	0.370	**–**0.041	–3.428 to 2.743	0.822			
Mean SBP (mmHg)	–0.144	–0.827 to 0.361	0.430	**–**0.183	–0.929 to 0.310	0.316			
Mean DBP (mmHg)	–0.009	–1.088 to 1.037	0.961	**–**0.083	–1.361 to 0.862	0.650			
LDL (mg/dL)	0.111	–0.230 to 0.426	0.546	**–**0.058	–0.399 to 0.292	0.754			
Serum uric acid (mg/dL)	–0.447	–15.438 to –2.240	0.100	**–**0.264	–12.936 to 1.997	0.145			
Serum albumin (g/L)	–0.060	–1.541 to 1.112	0.743	–0.176	–1.514 to 1.273	0.861			
Initial htTKV (mL/m)	–0.510	–0.043 to –0.010	0.003*	**–**0.602	–0.049 to 0.017	< 0.001*	**–**0.551	**–**0.045 to **–**0.015	< 0.001*
24-hr urine protein (mg/day/1.73 m^2^)	–0.326	–0.097 to –0.004	0.068	**–**0.399	–0.111 to –0.009	0.024*	**–**0.157	–0.096 to 0.049	0.513
24-hr urine albumin (mg/day/1.73 m^2^)	–0.332	–0.153 to 0.004	0.063	**–**0.410	–0.176 to –0.016	0.020*	**–**0.021	–0.112 to 0.122	0.932

* indicates statistical significance (*P* < 0.05)

*eGFR* estimated glomerular filtration rate; *mGFR* measured glomerular filtration rate; *BMI* body mass index; *SBP* systolic blood pressure; *DBP* diastolic blood pressure; *LDL* cholesterol, low density lipoprotein cholesterol; *htTKV* height-adjusted total kidney volume

**Table 3:** The association of the percentage change in estimated GFR per year (%ΔeGFR/y) and the percentage change in measured GFR per year (%ΔmGFR/y) on different clinical parameters

Clinical parameters (*N* = 32)	%ΔeGFR/y			%ΔmGFR/y					
	**Univariate analysis**			**Univariate analysis**			**Multivariate analysis**		
	*r*	95 % CI	*p*	*r*	95 % CI	*p*	*r*	95 % CI	*p*
Age (year)	–0.082	**–**0.173 to 0.111	0.656	**–**0.100	–0.165 to 0.095	0.587			
BMI (Kg/m^2^)	–0.194	–0.863 to 0.266	0.288	**–**0.137	–0.715 to 0.328	0.454			
Mean SBP (mmHg)	0.093	–0.088 to 0.146	0.612	**–**0.148	–0.149 to 0.064	0.418			
Mean DBP (mmHg)	0.052	–0.179 to 0.236	0.778	**–**0.131	–0.255 to 0.122	0.476			
Initial eGFR (mL/min/1.73 m^2^)	0.385	0.008 to 0.142	0.030*	-	-	-			
Initial mGFR (mL/min/1.73 m^2^)	**-**	-	-	0.476	0.025 to 0.137	0.006*	0.265	–0.013 to 0.103	0.123
Initial htTKV (mL/m)	0.126	–0.002 to 0.005	0.493	**–**0.282	–0.006 to 0.001	0.117			
24-hr urine protein (mg/day/1.73 m^2^)	–0.301	–0.018 to 0.002	0.094	**–**0.419	–0.019 to –0.002	0.017*	0.038	–0.012 to 0.014	0.881
24-hr urine albumin (mg/day/1.73 m^2^)	–0.205	–0.025 to 0.007	0.261	**–**0.497	–0.033 to –0.007	0.004*	**–**0.357	**–**0.027 to **–**0.001	0.034*
%ΔhtTKV/y (%/year)	–0.254	–0.767 to 0.133	0.161	**–**0.501	–0.941 to –0.203	0.004*	**–**0.363	**–**0.791 to **–**0.039	0.032*

* indicates statistical significance (*P* < 0.05)

*%ΔeGFR/y* percentage change in estimated GFR per year, *%ΔmGFR/y* percentage change in measured GFR per year; *BMI* body mass index; *SBP* systolic blood pressure; *DBP* diastolic blood pressure; *htTKV* height-adjusted total kidney volume; *%ΔhtTKV/y* percentage change in height-adjusted total kidney volume per year

### The association between clinical parameters and initial mGFR

Since we found that mGFR had a better association with clinical parameter than eGFR did (Tables 2 and 3), we decided to use mGFR to represent renal function of the patients throughout the studies.

Using Pearson’s correlation coefficients, the study demonstrated that initial mGFR was significantly inversely correlated with 4 clinical parameters, including age (*r* = –0.427, *p* = 0.015), initial htTKV (*r* = –0.602, *p* < 0.001), 24-hr urine protein (*r* = –0.399, *p* = 0.024), and 24-hr urine albumin (*r* = –0.410, *p* = 0.020) as shown in Table 2. It should be noted that when using eGFR instead of mGFR, the association between initial eGFR and 2 clinical parameters including, 24-hr urine protein and 24-hr urine albumin were not statistically significant (Table 2).

Further investigation by using multivariate regression analysis revealed that initial mGFR was significantly, independently, and inversely correlated only with initial htTKV (*p* < 0.001) and age (*p* = 0.016), Table 2.

**Fig. 3:**
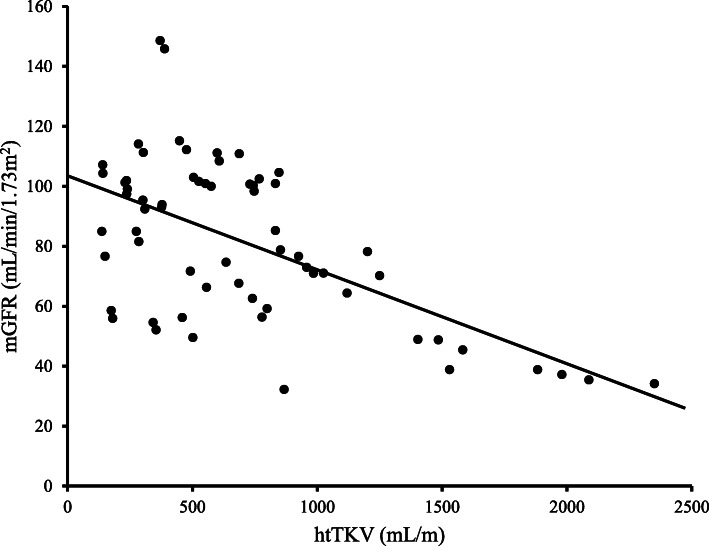
The relationship between mGFR and htTKV in 32 ADPKD patients over the study period using all data points of each patient. mGFR is significantly inversely correlated with htTKV (*y* = –0.032*x* + 104.06, *r* = –0.599, *p* < 0.001). mGFR; mearsured glomerular filtration rate, htTKV; height-adjusted total kidney volume

Figure 3 demonstrates the relationship between mGFR and htTKV in 32 ADPKD patients over the study period using all data points of each patient. It showed that mGFR was significantly inversely correlated with htTKV. The equation was as follows: *y* = –0.032*x* + 104.06, *r* = –0.599, *p* < 0.001.

### The association between clinical parameters and the percentage change in mGFR per year

The %ΔmGFR/y was significantly correlated with initial mGFR (*r* = 0.476, *p* < 0.006) and inversely correlated with other 3 clinical parameters, including 24-hr urine protein (*r* = –0.419, *p* = 0.017), 24-hr urine albumin (*r* = –0.497, *p* = 0.004) and %ΔhtTKV/y (*r* = –0.501, *p* = 0.004) as shown in Table 3. When using eGFR instead of mGFR, the association between the %ΔeGFR/y and 3 clinical parameters including, 24-hr urine protein, 24-hr urine albumin, and %ΔhtTKV/y were not statistically significant (Table 3).

Further multivariate analysis showed that the %ΔmGFR/y was significantly, independently, and inversely correlated only with the %ΔhtTKV/y (*p* = 0.032) and 24-hr urine albumin (*p* = 0.034), Table 3.

**Fig. 4:**
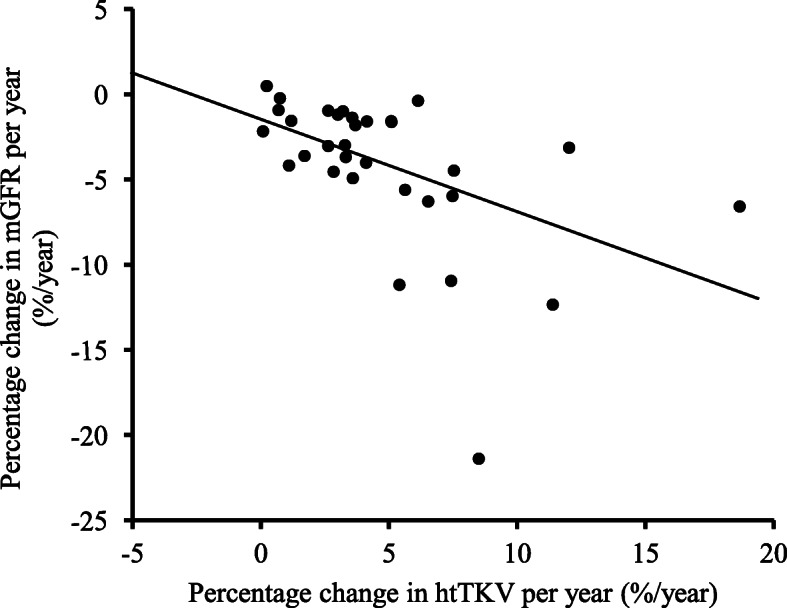
The relationship between the percentage change in mGFR per year (%ΔmGFR/y) and the percentage change in htTKV per year (%ΔhtTKV/y) in 32 ADPKD patients over the study period. The %ΔmGFR/y is significantly inversely correlated with the %ΔhtTKV/y (*y* = –0.572*x* – 1.486, *r* = –0.501, *p* = 0.004.). mGFR; measured glomerular filtration rate, htTKV; height-adjusted total kidney volume; %ΔmGFR/y, percentage change in mGFR per year; %ΔhtTKV/y, percentage change in htTKV per year

Figure 4 demonstrates the relationship between the %ΔmGFR/y and the %ΔhtTKV/y. The %ΔmGFR/y was significantly inversely correlated with the %ΔhtTKV/y. The equation was as follows: *y* = –0.572*x* – 1.486, *r* = –0.501, *p* = 0.004.

For purpose of clinical application, univariate regression analysis was utilized to study the relationship between the %ΔmGFR/y and the %ΔhtTKV/y. It was found that there was an association between a 5 % change in %ΔhtTKV/y and a 5 % change in %ΔmGFR/y. In this study, no patients who had a %ΔhtTKV/y of less than 5 % developed a %ΔmGFR/y of more than 5 %. By contrast, patients who had a %ΔhtTKV/y of more than or equal to 5 % had a high prevalence (61.5 %) to develop a %ΔmGFR/y of more than 5 %, *p* = 0.003.

In the case of albuminuria, the ROC curve was used to find the cutting point of 24-hr urine albumin to predict the change in %ΔmGFR/y of 5 %. We found that 24-hr urine albumin at 30 mg/day had a sensitivity of 62.5 % and specificity of 79.2 % to predict this change. In this study, patients who had a 24-hr urine albumin of less than 30 mg/day/1.73 m^2^ were less likely (13.0 %) to develop a %ΔmGFR/y of more than 5 %. On the other hand, patients who had a 24-hr urine albumin more than or equal to 30 mg/day/1.73 m^2^ had a higher chance (55.6 %) to develop a %ΔmGFR/y of more than 5 %, *p* = 0.023.

### The association between clinical parameters and the percentage change in htTKV per year (%ΔhtTKV/y)

The %ΔhtTKV/y was significantly correlated with initial htTKV (*r* = 0.420, *p* = 0.017) and 24-hr urine albumin (*r* = 0.384, *p* = 0.030) as shown in Table 4. Multivariate regression analysis showed that %ΔhtTKV/y was significantly and independently correlated only with htTKV (*p* = 0.017), Table 4. The equation was as follows: *y* = 0.003*x* + 0.429, *r* = 0.420, *p* = 0.017.

**Table 4:** The association of the percentage change in height-adjusted total kidney volume per year (%ΔhtTKV/y) and different clinical parameters

Clinical parameters (*N* = 32)	%ΔhtTKV/y					
	**Univariate analysis**			**Multivariate analysis**		
	*r*	95 % CI	*p*	*r*	95 % CI	*p*
Age (year)	0.073	**–**0.092 to 0.136	0.692			
BMI (Kg/m^2^)	0.096	–0.340 to 0.578	0.600			
Mean SBP (mmHg)	0.115	–0.064 to 0.122	0.532			
Mean DBP (mmHg)	0.324	–0.013 to 0.302	0.071			
Initial eGFR (mL/min/1.73 m^2^)	**–**0.158	–0.082 to 0.033	0.387			
Initial mGFR (mL/min/1.73 m^2^)	**–**0.333	–0.102 to 0.003	0.062			
Initial htTKV (mL/m)	0.420	0.001 to 0.006	0.017*	0.420	0.001 to 0.006	0.017*
24-hr urine protein (mg/day/1.73 m^2^)	0.349	0.000 to 0.016	0.050			
24-hr urine albumin (mg/day/1.73 m^2^)	0.384	0.001 to 0.026	0.030*	0.219	–0.006 to 0.022	0.276

* indicates statistical significance (*P* < 0.05)

*BMI* body mass index; *SBP* systolic blood pressure; *DBP* diastolic blood pressure; *eGFR* estimated glomerular filtration rate; *mGFR* measured glomerular filtration rate; *htTKV* height-adjusted total kidney volume; *%ΔhtTKV/y* percentage change in height-adjusted total kidney volume per year

### The effect of ARBs/ACE inhibitors on clinical parameters

In this study, there were 19 patients using at least one ARB or ACE inhibitor for treatment of hypertension. The other 13 (5 with hypertension on calcium channel blockers and 8 without hypertension) patients did not use ARBs or ACE inhibitors. Clinical parameters of ADPKD patients using ARBs/ACE inhibitors versus without ARBs/ACE inhibitors were shown in Table 5. There was no difference in blood pressure between the two groups. However, the mean 24-hr urine albumin was significantly lower in patients using ARBs/ACE inhibitors group (38.2 ± 57.4 vs. 131.8 ± 146.1, mg/day/1.73 m^2^, *p* = 0.045). Likewise, the mean 24-hr urine protein also had a tendency to be lower in patients using ARBs/ACE inhibitors group (150.5 ± 81.6 vs. 285.8 ± 237.6, mg/day/1.73 m^2^, *p* = 0.069), although this did not reach a statistically significant *p*-value. In addition, the mean 24-hr urine sodium tended to be higher in patients using ARBs/ACE inhibitors group (150.7 ± 25.9 vs. 135.2 ± 20.4, mEq/day, *p* = 0.070), but also not statistically significant. The %ΔhtTKV/y was not significantly different between the two groups (4.62 ± 3.43 vs. 4.98 ± 4.63, %/year, *p* = 0.812), and neither was the %ΔmGFR/y (–4.03 ± 3.59 vs. –4.48 ± 5.63, %/year, *p* = 0.804).

**Table 5:** Clinical parameters of patients on ARBs/ACE inhibitors versus without ARBs/ACE inhibitors

Clinical parameters	On ARBs/ACE inhibitors (*N* = 19)	No ARBs/ACE inhibitors (*N* = 13)	*P* Value
SBP (mmHg)	130.9 ± 15.6	130.2 ± 15.9	0.908
DBP (mmHg)	79.5 ± 9.1	78.8 ± 8.5	0.825
24-hr urine protein (mg/day/1.73 m^2^)	150.5 ± 81.6	285.8 ± 237.6	0.069
24-hr urine albumin (mg/day/1.73 m^2^)	38.2 ± 57.4	131.8 ± 146.1	0.045*
24-hr urine sodium (mEq/day)	150.7 ± 25.9	135.2 ± 20.4	0.070
%ΔhtTKV/y (%/year)	4.62 ± 3.43	4.98 ± 4.63	0.812
%ΔmGFR/y (%/year)	–4.03 ± 3.59	–4.48 ± 5.63	0.804

Data presented as mean ± SD

* indicates statistical significance (*P* < 0.05)

*ARB* angiotensin receptor blocker; *ACE inhibitor* angiotensin converting enzyme inhibitor; *SBP* systolic blood pressure; *DBP* diastolic blood pressure; *%ΔhtTKV/y* percentage change in height-adjusted total kidney volume per year; *%ΔmGFR/y* percentage change in measured GFR per year

## Discussion

Evaluation of renal function is a crucial part in assessing kidney disease, especially in ADPKD patients whose kidney functions remain stable for years, followed by a sharp decline in GFR once a critical renal size is reached [6, 7]. Assessment of renal function by estimated GFR using CKD-EPI and MDRD has been shown to have wide and unpredictable deviation of estimations compared to the measured GFR changes in ADPKD patients [32]. The authors in the former study stated that one-year GFR changes estimated by both prediction formulas failed to correlate, to an appreciable extent, with measured changes and suggested that these surrogate outcome variables were not appropriate to assess progression of ADPKD and response to treatment in research and clinics. In our studies, we also reaffirmed these results - the measured GFR by iohexol had a better correlation with clinical parameters compared to eGFR (CKD-EPI). Thus, in this study we used the mGFR by iohexol to represent renal function in our cohort of ADPKD patients. From our understanding, this is the first study to evaluate renal function by measured GFR using iohexol in an Asian ADPKD population.

The mGFR change in ADPKD patients in our studies was –2.99 mL/min/1.73m^2^/year, which was comparable to GFR change measured by iothalamate clearance –2.79 mL/min/1.73m^2^/year in the US population [33]. This is likely due to the initial GFR (this study 83.8 VS US 91.4 mL/min/1.73m^2^) and initial TKV (this study 1083 VS US 1193 mL) being comparable between our and the US population. When compared with the Japanese ADPKD population, the estimated GFR change per year in Japanese ADPKD was –2.8 mL/min/1.73m^2^/year by using the MDRD Japanese equation [34]. The GFR change in our studies was also comparable to the Japanese population. However, the initial estimated GFR of 55.3 mL/min/1.73m^2^ and initial TKV of 1595 mL in Japanese studies were quite different to our population. This discrepancy in results may be explained by the difference in method used to evaluate GFR between the Japanese and our population (estimated GFR by Japanese MDRD equation and measured GFR by iohexol).

The calculated annual kidney growth rate in our study was 4.8 % (baseline TKV of 1083 mL) which was in agreement with the previous reported of 4 % with baseline TKV of 1194 mL [33], and 5.2 % with baseline TKV of 1060 mL [14]. In addition, the results of our studies showed that the percentage change in htTKV per year was significantly correlated solely with initial htTKV (Table 4). This finding was similar to the results of previous studies which reported that the baseline total kidney volume predicted the subsequent rate of increase in volume [14], Because renal growth is a continuous and relatively constant process, patients with the largest kidneys at a certain age should have the fastest rate of kidney enlargement. The results of CRISP have shown that the kidney growth occurs in an exponential fashion and the growth of kidneys in patients with ADPKD is primarily the result of growth of cysts. ADPKD disease progression was due to the increasing in size of an extremely large number of renal cysts, which causes enlargement of the whole kidney [14, 35, 36]. The larger kidneys are likely to be associated with more complications such as hypertension, pain, hematuria, hemorrhagic cysts, infected cysts, and the decline in renal function.

The relationship between the total kidney volume and renal function was well demonstrated in our studies (Table 2 and Fig. 3). Our studies have shown that the initial mGFR is significantly, independently, and inversely associated with patient’s age and initial htTKV and the latter has a stronger association. Our results were similar with the results reported in previous studies that showed the inverse correlation of kidney volume and renal function [14, 36]. Moreover, our studies also demonstrated that the percentage change of mGFR per year was significantly, independently, and inversely associated with the percentage change in height-adjusted total kidney volume per year and 24-hr urine albumin (Table 3 and Fig. 4). The inverse association between the change in renal function and the change in total kidney volume was well demonstrated. Several studies have shown that the rate of increase in TKV were highly variable and that patients with high rates of growth were more likely to suffer serious declines in GFR than those who showed a slower growth rate [14, 36, 37]. The results of our studies also demonstrated that there was an association between a 5 % change in TKV per year and a 5 % change in the mGFR per year. In our population, no patients who had the TKV change of less than 5 % per year developed the mGFR change of more than 5 % per year. By contrast, patients who had a TKV change of more than or equal to 5 % per year had a high prevalence to develop mGFR changes of more than 5 % per year. The sequential measurement of total kidney volume and cyst volume has been suggested as a surrogate marker of disease progression [14, 35–37]. Our findings were in harmony with previous studies.

Albuminuria is well known to be strongly associated with progression in glomerular diseases. A number of studies have shown that in patients with ADPKD, which was classified as a non-glomerular disease, renal progression is also associated with albuminuria [8, 33, 34]. A previous study showed that overt proteinuria (protein > 300 mg/day) was associated with large renal volumes, higher blood pressures, and low creatinine clearances in patients with ADPKD [8]. The studies of ADPKD progression by mGFR using iothalamate clearance showed that patients with greater proteinuria had a significantly larger increase in renal volume and decline in renal function than those with less proteinuria [33]. The findings in our studies that showed the GFR change was significantly, independently, and inversely correlated with 24-hr urine albumin (Table 3) coordinated with the results in previous studies [33]. In addition, our finding by univariate but not by multivariate analysis also suggested that the percentage change in htTKV per year was significantly correlated with 24-hr urine albumin (Table 4). In our population, we found that 24-hr urine albumin at 30 mg/day/1.73 m^2^ had a good prediction for a 5 % change in GFR per year. Patients who had 24-hr urine albumin of less than 30 mg/day/1.73 m^2^ were less likely to develop GFR change of more than 5 % per year. On the other hand, patients who had a 24-hr urine albumin more than or equal to 30 mg/day/1.73 m^2^ had a higher prevalence of developing GFR change of more than 5 % per year.

In this study, the mean 24-hr urine albumin in patients using ARBs/ACE inhibitors was lower than that of patients without ARBs/ACE inhibitors. However, there was no statistically significant difference in the %ΔhtTKV/y and %ΔmGFR/y between the two groups. The benefits of slowing kidney volume progression did not occur in patients using ARBs/ACE inhibitors in this study. This could be explained by a considerable number of non-hypertensive patients (8/13) in the non ARBs/ACE inhibitors group who may have a better prognosis in the changes of TKV or GFR than hypertensive patients. In addition, the study period may be too short to claim the beneficial effects of these drugs.

Some potential limitations in our study should be acknowledged. Due to the small number of participants, our trial may have had inadequate power to demonstrate the statistical significance of some clinical correlations. Another limitation is the technique used to quantify TKV which we manually measured on the MRI. This gives rise to more variation and inconsistency when compared to other automatic techniques. However, this method was preferred over other techniques such as the fully automated segmentation technique as enlarged ADPKD cysts tend to distort the surrounding anatomical structures of the kidneys, causing differentiation of the anatomical boundaries by automated segmentation almost unfeasible. Hopefully, in the future, a more accurate automated technique will be developed to assess TKV in ADPKD kidneys, allowing for a more consistent measurement.

## Conclusions

Our studies demonstrated that mGFR by iohexol plasma clearance is a more reliable and accurate method than eGFR for evaluating GFR changes in the early stages of patients with ADPKD. There is a strong inverse correlation between kidney volume and renal function assessed by mGFR in an Asian ADPKD population. The initial htTKV is a good predictor for kidney volume progression. The %ΔhtTKV/y is a good early surrogate marker for following up the decline in renal function. 24-hr urine albumin is also a good indicator for renal progression.

## Data Availability

The datasets generated and/or analyzed during the current study are not publicly available due to the institutional policy but are available from the corresponding author on reasonable request.

## Abbreviations

ADPKD: Autosomal dominant polycystic kidney disease
ESRD: End stage renal disease
eGFR: Estimated glomerular filtration rate
mGFR: Measured glomerular filtration rate
CKD-EPI: Chronic kidney disease epidemiology collaboration
MDRD: Modification of diet in renal disease
TKV: Total kidney volume
htTKV: Height-adjusted TKV
ΔhtTKV/y: Change in htTKV per year
%ΔhtTKV/y: Percentage change in htTKV per year
ΔeGFR/y: Change in eGFR per year
ΔmGFR/y: Change in mGFR per year
%ΔeGFR/y: Percentage change in eGFR per year
%ΔmGFR/y: Percentage change in mGFR per year
CRISP: Consortium of radiologic imaging studies of polycystic kidney disease
UPLC: Ultra performance liquid chromatography
